# Unveiling Photoperiod-Responsive Regulatory Networks in Tropical Maize Through Transcriptome Analysis

**DOI:** 10.3390/genes16020192

**Published:** 2025-02-04

**Authors:** Tianhui Zheng, Jinge Bo, Jing Wang, Siyuan Li, Haonan Li, Mengyao Liu, Hongbin Niu, Thanhliem Nguyen, Yanhui Chen, Juan Sun

**Affiliations:** 1 College of Agronomy, Henan Agricultural University, Zhengzhou 450002, China; zhengtianhui@henau.edu.cn (T.Z.); chy9890@163.com (Y.C.); 2 The National Engineering Research Centre for Wheat, Henan Agricultural University, Zhengzhou 450002, China; 3 Faculty of Natural Sciences, Quy Nhon University, Quy Nhon 590000, Vietnam; nguyenthanhliem@qnu.edu.vn

**Keywords:** maize, photoperiodic sensitive, transcription regulation, circadian clock, protein–protein interaction

## Abstract

**Background/Objectives:** Maize (*Zea mays* L.), a crop of worldwide importance, owes its adaptability to diverse environments to its genetic variation. However, tropical maize exhibits intrinsic photoperiod sensitivity, limiting its adaptability to temperate regions. Photoperiod sensitivity significantly affects the flowering time and other agronomic traits, but the underlying molecular mechanisms remain poorly understood. In this study, the aim is to elucidate the transcriptional regulatory networks mediating photoperiod responses in tropical maize inbred line Su65, providing insights into improving photoperiod adaptability. **Methods:** RNA-seq analysis was carried out to investigate photoperiod-responsive genes and pathways in tropical line Su65 exposed to varying photoperiod conditions. Differential expression analysis, functional enrichment, and the construction of protein–protein interaction (PPI) networks were carried out to investigate transcriptional dynamics. Additionally, qRT-PCR was employed to confirm the expression patterns of key candidate genes and generate detailed temporal expression profiles. **Results:** A total of 1728 differentially expressed genes (DEGs) were identified, with significant enrichment in pathways such as stress responses, redox homeostasis, and secondary metabolite biosynthesis. A set of new key hub genes (such as *Zm00001d048531*, *Zm00001d018821*, *Zm00001d034892*, etc.) were identified through PPI network analysis. Temporal expression profiling of *ZmPHYB1*, *ZmPHYC1*, *ZmFKF2*, *ZmGI2*, and *ZmPRR37a*, the key genes involved in circadian rhythms, revealed distinct regulatory patterns of photoperiod-sensitive genes at different time points, highlighting their roles in flowering time regulation and developmental transitions. **Conclusions:** In this study, critical molecular networks underlying photoperiod sensitivity in tropical maize are uncovered and a foundation is provided for improving photoperiod adaptability through genetic improvement. By integrating RNA-seq and qRT-PCR, the research offers valuable insights into transcriptional dynamics and their role in maize development under photoperiodic regulation.

## 1. Introduction

Maize (*Z. mays* L.), an important crop worldwide, serves as a major resource as a key provider of food, animal feed, and raw materials for various industries [1]. The genetic diversity of maize allows it to adapt to a wide range of environments, making it a versatile and economically significant crop. Maize originally evolved in tropical regions and has undergone extensive domestication over centuries, leading to the development of the two major germplasm groups of tropical and temperate maize [2]. This prolonged artificial selection has resulted in the specialization of germplasm pools adapted to distinct photoperiodic and climatic conditions.

Modern maize breeding programs have significantly reduced the genetic diversity of available germplasm due to an overemphasis on yield and other utilitarian traits [3]. The lack of genetic diversity presents challenges in addressing emerging agricultural and environmental stresses [4,5]. The integration of tropical and temperate maize germplasms holds great potential for improving key agronomic traits, such as stress resistance and adaptability to changing environments [3,6]. However, this approach is limited by the intrinsic sensitivity of tropical maize to photoperiod changes, which affects its adaptability to temperate regions [7,8].

Photoperiod signals play a crucial role in regulating the flowering time in plants. The molecular mechanisms underlying photoperiod regulation of heading date have been extensively studied in rice, a short-day model plant, which shares a similar photoperiodic response with maize. Under long-day conditions, photoperiod-sensitive key genes such as *Grain Number*, *Plant Height and Heading Date 7* (*Ghd7*) [9], *DTH8* [10], *Pseudo Response Regulator 37* (*PRR37*) [11], and *Early Heading Date 2* (*Ehd2*) [12] delay the heading date by repressing the expression of heading date active genes *Ehd1* [13] and *Hd3a* [14]. In contrast, the expression of these photoperiod-sensitive genes is significantly reduced under short-day conditions. These results suggest that the regulation of heading date in rice by photoperiod signals may primarily occur through the expression modulation of photoperiod-sensitive genes. Therefore, elucidating the transcriptional regulatory networks responding to photoperiod signals in plants is essential for understanding the molecular mechanisms of photoperiodic regulation of plant development. However, the regulatory network for the photoperiod control of maize development remains unclear.

In maize, photoperiod sensitivity greatly influences critical traits such as flowering time, plant height, number of leaves, and internode features [3,12,13,14]. Photoperiod sensitivity in maize has been widely studied, primarily focusing on its effects on flowering time, which is closely linked to grain formation and yield [8,15]. Studies have explored the chromosomal regions associated with photoperiod sensitivity [3,15,16,17,18] and several photoperiod-responsive genes were cloned, such as *ZmPHYB1/2* [19], *ZmPHYC1/2* [20], *ZmCCT9/10* [21,22], *ZmCCA1* [23], and *ZmPRR37a* [24], which regulate the flowering time in a photoperiodic dependent manner. Despite these advances, the underlying molecular networks that integrate photoperiod signals and developmental regulation remain insufficiently understood. While previous studies have predominantly concentrated on the genetic and phenotypic effects of photoperiod changes, our research seeks to explore the transcriptional regulatory networks underlying these responses in tropical maize.

RNA sequencing (RNA-seq) has become a powerful tool for exploring the transcriptional dynamics underlying photoperiod sensitivity and its effects on maize development [25,26]. RNA-seq analyses have identified genes participating in photoperiod-regulated mechanisms, such as those governing photosynthesis, phenylalanine biosynthesis, phosphate ion dynamics, and the regulation of circadian rhythms [26]. These investigations have shed light on the molecular mechanisms impacted by photoperiod variations; nonetheless, many primarily compare different inbred lines instead of examining temporal gene expression changes within one genotype.

Addressing the gaps in understanding molecular mechanisms linking photoperiod signaling and development, in this research, RNA-seq is applied to assess the transcriptional changes in photoperiod-sensitive genes in tropical maize. By performing differential expression profiling, functional enrichment analysis, and constructing protein interaction networks, we aim to reveal the pivotal genes and pathways contributing to photoperiod adaptation. Furthermore, temporal expression profiling will provide insights into how photoperiod signals regulate critical developmental transitions, such as flowering time. This research not only deepens our understanding of the molecular mechanisms underlying photoperiod sensitivity but also provides valuable resources for breeding programs aimed at improving photoperiod adaptability and agronomic performance in maize.

## 2. Materials and Methods

### 2.1. Plant Materials and Growth Conditions

The seedlings of tropical maize inbred line Su65, which were reported in previous research [27], were cultivated in conventional soil under artificial long day (LD) (light/darkness: 16 h/8 h at 30 °C) and short day (SD) (light/darkness: 8 h/16 h at 30 °C) photoperiods in climatic chambers at 60% humidity. The light was provided by fluorescent white-light tubes (16000 lux). Plants were grown until the sixth true leaf stage emerged, and sampling was performed at this stage.

**Figure 1:**
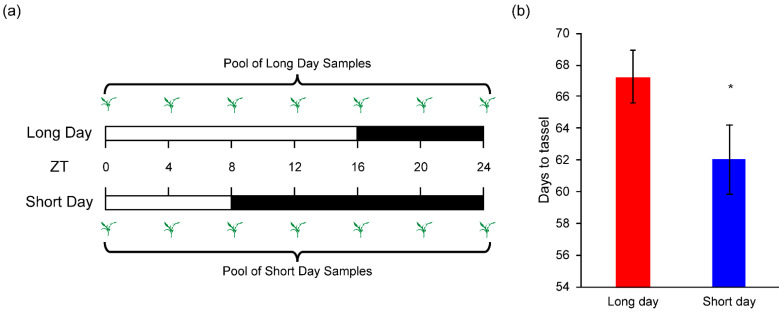
Sample collection and photoperiodic sensitivity of Su65. (**a**) Schematic representation of sample collection; (**b**) effects of photoperiod treatments on flowering time. Error bars indicate SD (*n* = 20), and asterisks denote significant differences (*p* < 0.05) between treatments.

To comprehensively analyze gene expression patterns, leaf samples were collected at the following seven time points: ZT0, ZT4, ZT8, ZT12, ZT16, ZT20, and ZT24, where ZT represents ’zeitgeber time’ (time since lights on in the controlled environment). ZT0 corresponds to the start of the light period, and subsequent sampling points were spaced at 4 h intervals, covering a full 24 h photoperiod cycle (Figure 1a). Samples from all time points within each photoperiod were pooled to create a composite sample representing the 24 h cycle. Three biological replicates were prepared by repeating the sampling process three times independently for each photoperiod condition.

### 2.2. RNA Extraction, Library Construction, and Sequencing

RNA was extracted from samples with TRIzol (Invitrogen, Carlsbad, CA, USA), and mRNA was enriched using Oligo (dT) beads. Ribosomal RNA was depleted via the Ribo-Zero™ Magnetic Kit (Epicentre, Madison, WI, USA). Fragmented mRNA was converted into cDNA; the first-strand synthesis utilized random primers, while second-strand synthesis was carried out with DNA polymerase I and RNase H. Double-stranded cDNA was purified, end-repaired, polyadenylated, and ligated to Illumina adapters. Following size selection and PCR amplification, libraries were sequenced on the Illumina HiSeq 2500 platform for high-throughput data generation.

### 2.3. Bioinformatics Analysis

#### 2.3.1. Filtering of Clean Reads and Removal of Ribosomal RNA

To ensure high-quality sequencing data for downstream analyses, raw reads obtained from sequencing were pre-processed to eliminate low-quality sequences and adapter contamination. The filtering process was conducted using fastp [28] (version 0.18.0). To eliminate rRNA contamination, clean reads were aligned against an rRNA database using Bowtie2 [29] (version 2.2.8). Reads that matched rRNA sequences were removed.

#### 2.3.2. Genome Alignment and Gene Expression Quantification

Clean reads were aligned to the maize reference genome using HISAT2 (version 2.4) [30]. The alignment process facilitated accurate positioning of reads along the genome for subsequent transcript assembly and expression analysis. Gene expression levels were quantified using StringTie [31,32] (version 1.3.1) based on the reference-guided assembly approach. Expression levels were normalized and reported as FPKM values.

#### 2.3.3. Identification of Differentially Expressed Genes (DEGs)

To analyze differential expression, DESeq2 [33] was utilized for comparisons across groups, and edgeR [34,35] was used for pairwise analyses. Genes or transcripts with an FDR below 0.05 and an absolute fold change of at least 2 were classified as differentially expressed.

#### 2.3.4. Relationship Analysis of Samples

To assess the reliability and reproducibility of the experimental data, correlation analysis was conducted using R software (4.0.3). The consistency among biological replicates was assessed by calculating Pearson correlation coefficients and principal component analysis (PCA).

#### 2.3.5. Gene Ontology (GO) Enrichment Analysis

DEGs were mapped to GO terms in the Gene Ontology database http://www.geneontology.org (accessed on 10 October 2020). The frequency of each term among DEGs was calculated, and significantly enriched terms were identified using a hypergeometric test. The probability (*p*-value) for enrichment was calculated as follows:

P=1−∑i=0m−1 (Mi)(N−Mn−i)(Nn)

To control for multiple testing, *p*-values were adjusted using the false discovery rate (FDR), with FDR ≤ 0.05 considered statistically significant. GO terms that met this criterion were regarded as significantly enriched, enabling the identification of predominant biological functions associated with the DEGs.

#### 2.3.6. Pathway Enrichment Analysis

To identify significantly enriched pathways, DEGs were mapped to the Kyoto Encyclopedia of Genes and Genomes (KEGG) pathways, and the number of genes associated with each pathway was computed [36]. The hypergeometric test was used to determine whether specific pathways were overrepresented in the DEG set relative to the genome-wide background. The resulting *p*-values were adjusted for multiple testing using the false discovery rate (FDR) method, with FDR ≤ 0.05 considered statistically significant. Pathways satisfying this threshold were classified as significantly enriched, highlighting biological processes and signaling networks potentially impacted by differential gene expression.

#### 2.3.7. Hub Gene Identification Within PPI Networks

To identify central hub genes and key regulators within the constructed PPI networks, the CytoHubba plugin in Cytoscape was employed [37,38]. Hub gene selection was based on connectivity and network centrality metrics, utilizing the maximal clique centrality (MCC) algorithm. MCC scoring is computed as follows:

$$MCC(v)=\sum_{C\in S(v)}\;(|C|)$$

### 2.4. Quantitative Real-Time PCR Analysis

Complementary DNA (cDNA) was synthesized from total RNA using a reverse transcription kit (Vazyme, Nanjing, China) according to the manufacturer’s instructions. Quantitative real-time PCR (qRT-PCR) was performed with SYBR Premix Ex Taq™ II (Sangon Biotech, Shanghai, China) on a QuantStudio™ 5 system (Thermo Fisher Scientific, Waltham, MA, USA), following a three-step cycling protocol. Gene expression levels were normalized to the internal reference gene *ZmEf1α* (*Zm00001d046449*) and calculated using the 2^−ΔCT^ method. Primer sequences are provided in Table S1, and each sample was analyzed in 5 biological replicates for reliability.

## 3. Results

### 3.1. Samples Collection and Phenotype of Su65 Under Different Photoperiod

The tropical maize inbred line Su65 was grown under LD conditions until it developed six true leaves. Subsequently, plants were subjected to either LD or SD photoperiods for an additional 7 days. Following the photoperiod treatments, samples were collected as outlined in the Section 2 and illustrated in Figure 1a. After sample collection, all seedlings, regardless of their initial photoperiod treatment, were transferred to LD conditions to continue growth. The day to tassel was recorded (Figure 1b). Under LD conditions, the day to tassel was 67 days, while under SD conditions, it was 62 days. A significant increase was observed in maize flowering due to the artificial LD treatment. This result indicate that Su65 showed significant photoperiodic sensitivity under LD conditions.

### 3.2. RNA Sequencing and Data Quality Assessment

#### 3.2.1. Sequencing Quality

A total of 45.38 Gb raw data were obtained from the transcriptome sequencing of six samples. Using fastp, raw data were filtered, and the data filtering statistics are shown in Table 1. Adapter contamination rates ranged from 0.02% to 0.03%, indicating effective adapter trimming. Low-quality reads accounted for only 0.10% to 0.16%, and polyA-containing reads were completely removed. Reads containing undetermined bases (N) were negligible at 0.01%. The percentage of clean reads retained after filtering was exceptionally high, ranging from 99.81% to 99.87%, ensuring data reliability for downstream analysis.

Following data filtering, sequencing quality analysis confirmed the high accuracy and reliability of the processed data (Table 2). The Q20 and Q30 scores consistently exceeded 98.5% and 95.2%, respectively, reflecting low base-calling error rates. The GC content ranged from 54.15% to 55.32%, closely matching the expected GC composition of the maize genome. These results demonstrate the overall quality and integrity of the sequencing data, ensuring its suitability for downstream transcriptome analyses.

To remove contaminating ribosomal RNA (rRNA) that typically constitutes the majority of RNA in biological samples, we performed rRNA filtering using Bowtie2 to align clean reads to the species-specific rRNA database. After removing the rRNA-mapped reads, the remaining unmapped reads were retained for subsequent transcriptome analysis. Table S2 summarizes the effect of rRNA removal, showing that the majority of reads were successfully aligned to the genome, with a minimal loss of data.

#### 3.2.2. Mapping Results

To ensure accurate transcript quantification and downstream analysis, clean reads were aligned to the maize reference genome Zm-B73-REFERENCE-GRAMENE-4.0 using HISAT2, and the mapping statistics are summarized in Table 3. Unique mapping rates ranged from 84.86% to 88.51%, while multiple mapping rates remained between 3.01% and 3.23%. The total mapping rates were consistently high, ranging from 87.87% to 91.59%, indicating accurate and efficient genome alignment. The proportion of unmapped reads was minimal, between 8.41% and 12.13%, reflecting low data loss and high mapping specificity, which supports the reliability of subsequent analyses.

#### 3.2.3. Principal Component Analysis (PCA)

**Figure 2:**
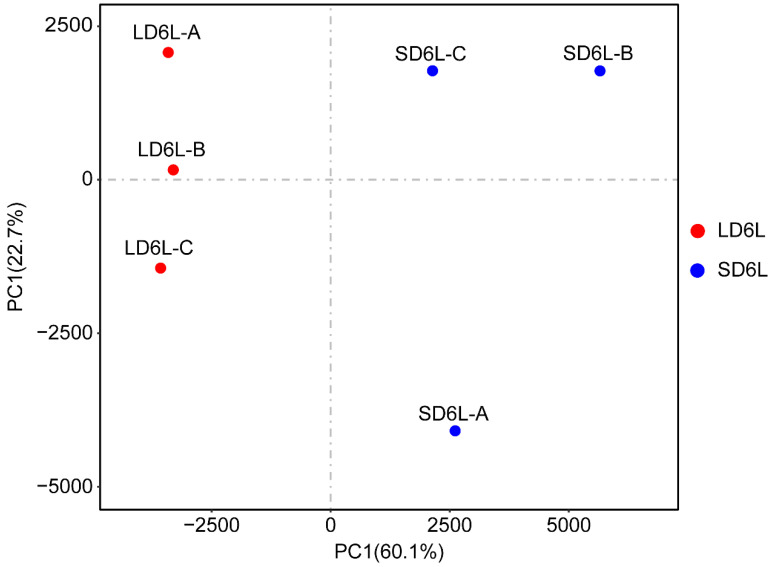
Principal component analysis (PCA) of gene expression profiles under LD and SD conditions.

To assess the sample clustering based on gene expression profiles, PCA was performed and is shown in Figure 2, and the results are summarized in Table S3. The first two principal components (PC1 and PC2) explained 60.1% and 22.7% of the total variance, respectively. Samples from LD and SD conditions were distinctly separated along these components, validating experimental conditions and demonstrating the consistency among biological replicates.

The combined results from the data quality assessment, read mapping statistics, and PCA analysis confirm the reliability and accuracy of the sequencing dataset. High clean read retention, efficient genome alignment, and clear sample separation highlight the robustness of the data. These findings provide a strong foundation for subsequent transcriptome analyses, ensuring meaningful and reliable biological interpretations.

### 3.3. Differential Gene Expression Analysis

To understand the molecular basis of maize photoperiod responses, we first identified the DEGs between LD and SD conditions. This allowed us to determine which genes were actively regulated under different photoperiods, forming the foundation for further functional analysis.

#### 3.3.1. Overall Differential Gene Statistics

**Figure 3:**
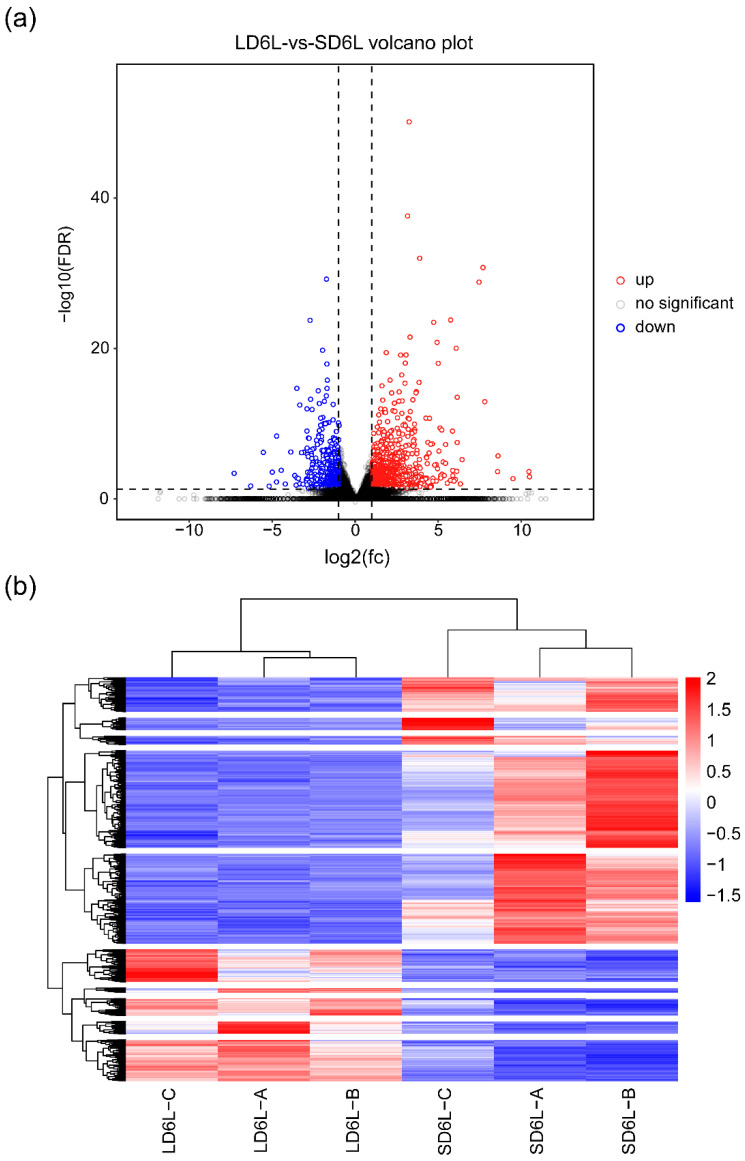
DEG analysis under LD and SD conditions. (**a**) Volcano plot showing DEGs between SD and LD conditions. Genes with significantly higher expression in SD compared to LD are labeled as “up” (red), while those with significantly lower expression are labeled as “down” (blue). Genes with no significant expression change are labeled as “no significant” (gray). Statistical significance is determined by FDR < 0.05 and |log_2_(Fold Change)| > 1; (**b**) heatmap of gene expression profiles under LD and SD conditions. “LD6L” indicates samples collected under long-day conditions, while “SD6L” indicates samples collected under short-day conditions. Gene expression levels are scaled by row and displayed using a red-to-blue color gradient, where red represents high expression and blue indicates low expression. Clustering was performed based on gene expression patterns.

Gene expression levels were analyzed using DESeq2 based on read count data. Genes with FDR < 0.05 and |log_2_FC| > 1 were considered significantly differentially expressed. To provide an intuitive overview of the DEG distributions, a volcano plot (Figure 3a) was generated, highlighting genes with significant expression changes. In the comparison between LD6L and SD6L samples, a total of 1728 DEGs were identified, including 1196 upregulated and 532 downregulated genes (Figure S1). Genes with higher |log_2_FC| values and lower FDRs are prominently located at the top edges of the plot, reflecting their strong differential expression.

Additionally, a heatmap (Figure 3b) was constructed to visualize hierarchical clustering based on DEG expression profiles. This heatmap reveals clear sample clustering, where LD6L are grouped together, as are SD6L. Furthermore, DEGs were clustered into distinct expression groups, highlighting genes with similar expression patterns across samples.

The hierarchical clustering analysis enabled the classification of DEGs into 10 clusters, reflecting contrasting gene expression trends between the two photoperiod treatments (Table S4). These clusters provide a valuable framework for prioritizing candidate genes for downstream validation and functional studies. By combining both statistical significance (FDR) and expression magnitude (log_2_FC), the analysis effectively identifies genes with biologically meaningful responses to photoperiod changes.

#### 3.3.2. Functional Enrichment Analysis

To elucidate the biological functions and pathways influenced by the DEGs, we performed GO and KEGG enrichment analyses.

1. GO Enrichment Analysis

**Figure 4:**
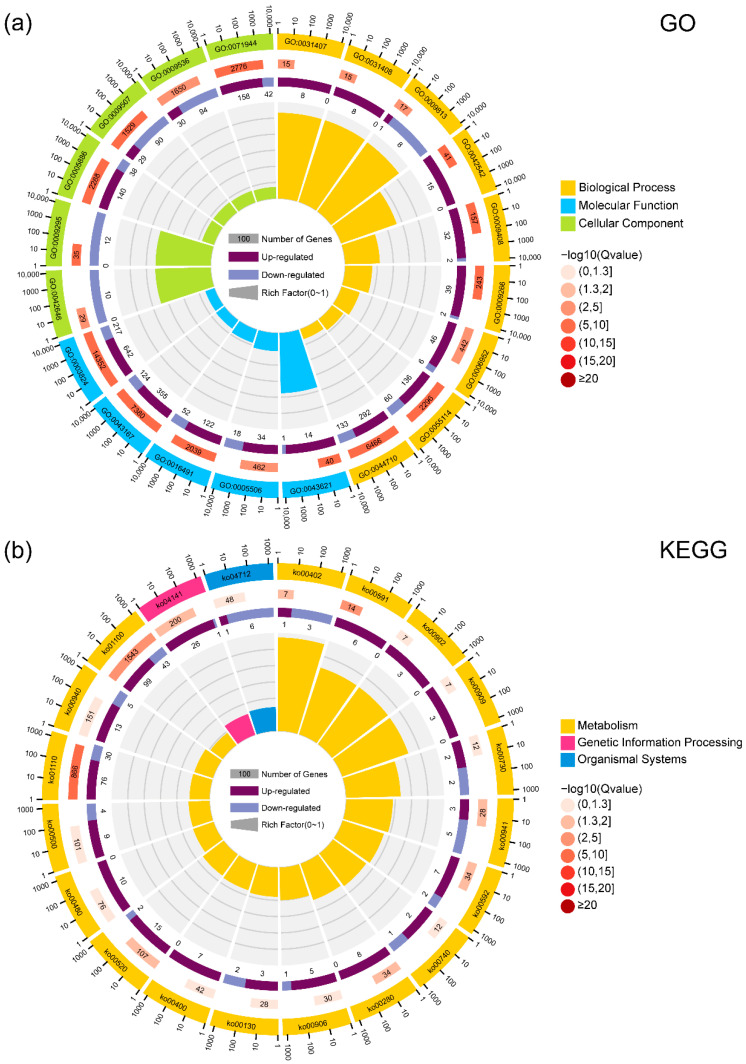
Functional enrichment analysis of DEGs. (**a**) GO enrichment analysis of the top 20 significantly enriched GO terms. The circular plot displays enriched GO terms grouped into the three main categories, as follows: biological process (BP), molecular function (MF), and cellular component (CC). The size of each section represents the number of DEGs associated with the corresponding GO terms, while color intensity reflects the enrichment significance; (**b**) KEGG pathway enrichment analysis of the top 20 significantly enriched pathways. The circular plot illustrates enriched pathways involved in various metabolic, signaling, and regulatory processes. The section size indicates the number of DEGs mapped to each pathway, while color intensity reflects the enrichment significance.

The GO enrichment analysis revealed several key biological processes, molecular functions, and cellular components associated with photoperiod-responsive genes (Figure 4a). Among the most significantly enriched terms were processes related to stress responses, including response to heat (GO:0009408) and response to hydrogen peroxide (GO:0042542), suggesting activation of protective mechanisms under environmental stress. Additionally, the terms oxidation-reduction process (GO:0055114) and oxidoreductase activity (GO:0016491) indicated the active regulation of redox balance and metabolic adjustments. Furthermore, pathways such as flavonoid biosynthetic process (GO:0009813) and iron ion binding (GO:0005506) highlighted the involvement of secondary metabolite biosynthesis and metal ion coordination, essential for maintaining cellular homeostasis and environmental adaptation.

2. KEGG Pathway Enrichment Analysis

The KEGG pathway analysis further supported the functional significance of DEGs by identifying pathways involved in essential metabolic and regulatory processes (Figure 4b). Prominent pathways such as metabolic pathways (ko01110) underscored the metabolic flexibility required for photoperiod adaptation. Fatty acid metabolic processes, including linoleic acid metabolism (ko00591) and α-linolenic acid metabolism (ko00592), were also enriched, reflecting lipid-based signaling and membrane dynamics. Additionally, pathways like phenylpropanoid biosynthesis (ko00940) and flavonoid biosynthesis (ko00941) emphasized roles in antioxidant production and stress mitigation. Notably, the identification of circadian rhythm-plant (ko04712) indicated the possible regulation of light-responsive gene networks linked to photoperiod-dependent flowering control.

Overall, the GO analysis highlighted biological processes essential for stress adaptation and metabolic regulation, while the KEGG analysis provided insights into key metabolic pathways and regulatory networks. Together, these results illuminate the intricate molecular mechanisms underlying photoperiod responses in maize.

### 3.4. Protein–Protein Interaction (PPI) Network Analysis

To further explore the functional relationships among DEGs, we performed a PPI network analysis. STRING http://string-db.org (accessed on 10 October 2020) was used to construct interaction networks based on known and predicted protein associations.

Differentially expressed genes were divided into upregulated and downregulated groups under short-day conditions. Each group was analyzed separately to identify interaction networks. The STRING database was queried to extract PPI data, including experimentally validated interactions, co-expression relationships, and predicted functional associations.

**Figure 5:**
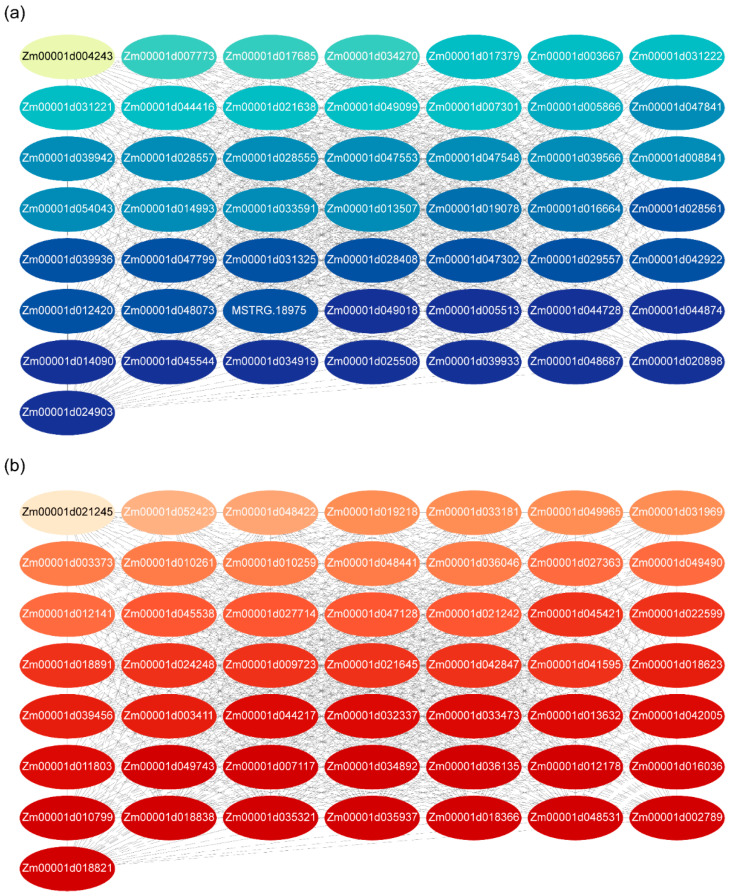
PPI networks of DEGs. (**a**) PPI network of the top 50 upregulated genes identified using the MCC algorithm in CytoHubba; (**b**) PPI network of the top 50 downregulated genes. Nodes represent proteins, and edges indicate interactions based on STRING confidence scores. Node colors represent degree centrality in the PPI network, with light-to-dark gradients (yellow/red or green/blue) indicating low-to-high connectivity.

Cytoscape was employed to visualize and analyze the networks. For each group, the CytoHubba plugin was used to calculate top-ranked genes based on the maximal clique centrality (MCC) algorithm. The top 50 genes with the highest MCC scores were selected to construct and visualize the PPI networks for both upregulated and downregulated genes (Figure 5).

The functional annotations and cluster classifications for the top 50 upregulated genes are shown in Table S5. These genes involve critical biological processes, including protein folding, stress responses, and metabolic regulation. Notably, many of the identified hub genes encode heat shock proteins and chaperones, which are essential for maintaining protein stability and cellular homeostasis under stress conditions.

For downregulated genes, Table S6 provides detailed annotations and clustering information. These genes are primarily associated with chloroplast and mitochondrial functions, including photosynthesis, RNA processing, and oxidative stress responses. Several hub genes, such as superoxide dismutase and chloroplast RNA processing factors, were identified, highlighting their potential roles in maintaining cellular redox balance and transcriptional regulation under photoperiod changes.

Overall, the PPI network analysis revealed distinct functional modules and hub genes associated with stress adaptation and metabolic processes. The identification of heat shock proteins and chloroplast-related factors emphasizes their involvement in photoperiod-regulated pathways. These findings provide a foundation for further experimental validation and functional characterization of key genes involved in photoperiodic responses and stress tolerance mechanisms.

### 3.5. Circadian Rhythm-Related Gene Expression Patterns Under Different Photoperiods

To further investigate the rhythmic expression patterns of circadian rhythm-related DEGs identified from RNA-seq data (gene id and gene symbol shown in Table S7), we performed qRT-PCR analysis to examine their transcriptional dynamics under LD and SD conditions over 24 h. This approach aimed to validate RNA-seq findings and explore photoperiod-induced changes in gene expression rhythms.

#### 3.5.1. Photoreceptor Genes Respond Differently to Photoperiod Changes

**Figure 6:**
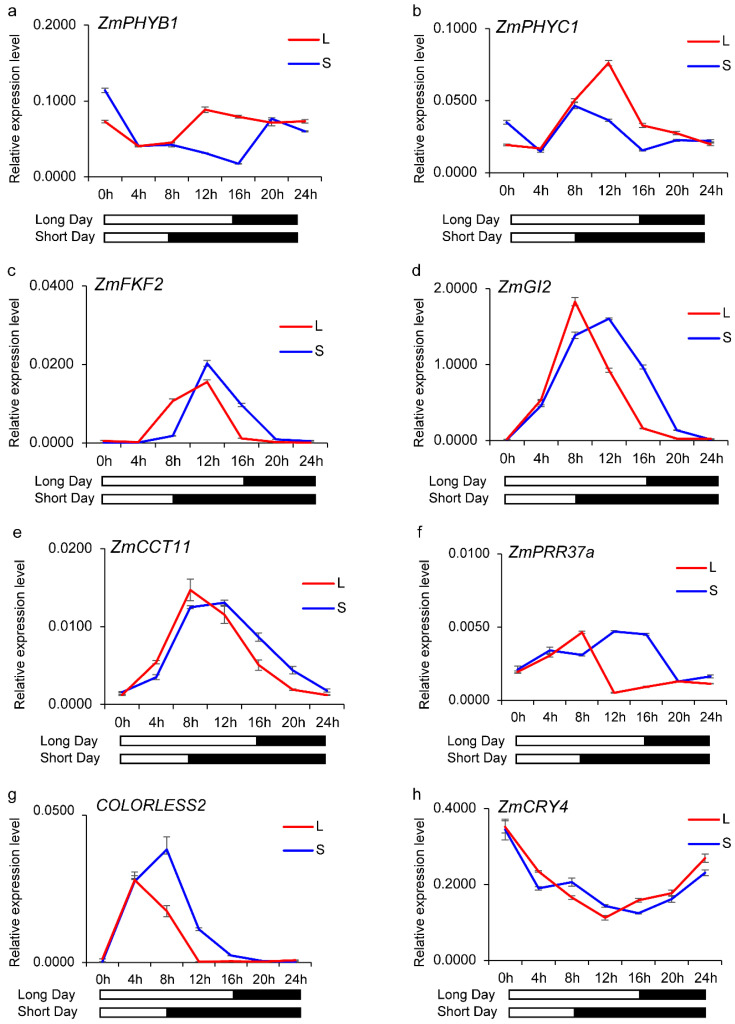
Expression patterns of circadian rhythm-related genes under LD and SD conditions. (**a**,**b**) Expression profiles of phytochrome genes *ZmPHYB1* and *ZmPHYC1*; (**c**–**g**) circadian clock genes (*ZmFKF2*, *ZmGI2*, *ZmCCT11*, *ZmPRR37a*, and *COLORLESS2*); (**h**) expression profiles of cryptochrome gene *ZmCRY4*. Error bars represent standard deviations.

The expression profiles of *ZmPHYB1* and *ZmPHYC1*, which encode phytochrome photoreceptors, displayed significant diurnal variations under SD conditions. Both genes exhibited higher transcript levels between 8 h and 20 h in LD compared to SD, suggesting enhanced photoperiod responsiveness under LD conditions (Figure 6a,b). In contrast, the cryptochrome gene *ZmCRY4* showed no significant differences in expression levels between LD and SD across the 24 h cycle, indicating a more stable expression pattern independent of photoperiod changes (Figure 6h).

#### 3.5.2. Circadian Clock Genes Exhibit Photoperiod-Dependent Phase Shifts

Several key circadian rhythm genes, including *ZmFKF2*, *ZmGI2*, *ZmCCT11*, *ZmPRR37a*, and *COLORLESS2*, exhibited rhythmic expression patterns in both LD and SD conditions, with distinct phase shifts (Figure 6c–e,g). Notably, the expression peaks under SD occurred significantly later than those observed under LD, reflecting a delay in circadian timing in response to extended darkness hours. Among these genes, *ZmFKF2* and *COLORLESS2* displayed slightly higher peak expression levels under SD than LD, despite the observed phase shifts (Figure 6c,g). These patterns suggest that photoperiod length modulates both the timing and amplitude of circadian gene expression, potentially influencing downstream developmental responses.

#### 3.5.3. Validation of Top-Ranked Downregulated Genes

**Figure 7:**
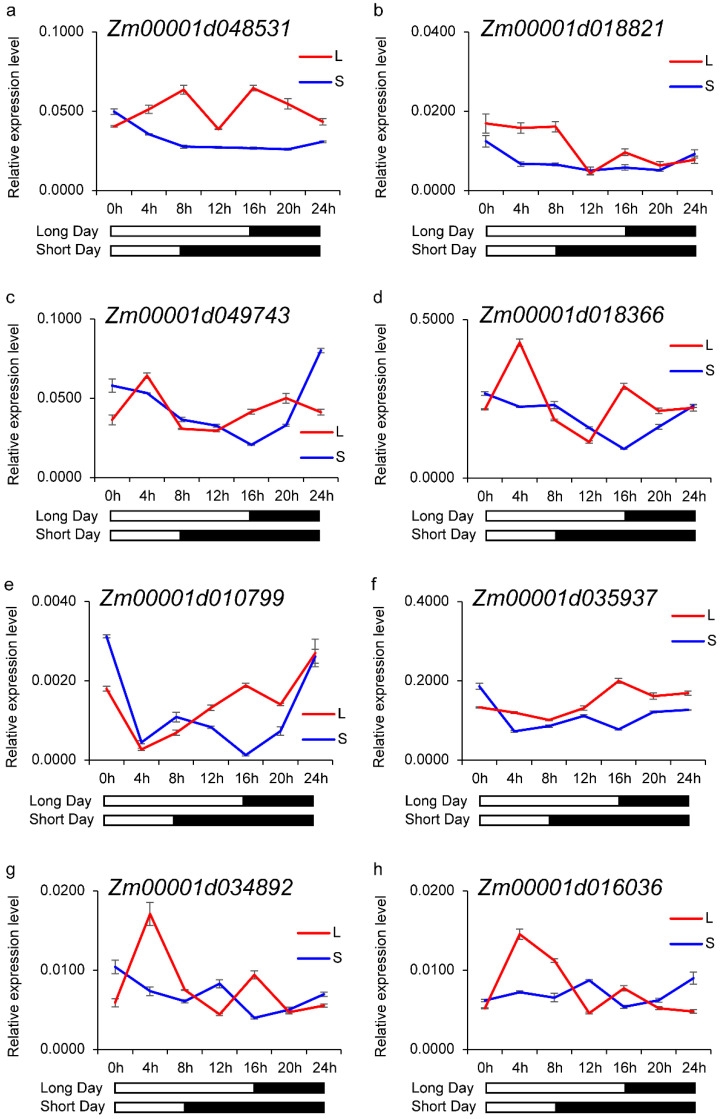
Expression profiles of selected downregulated genes identified by PPI network analysis. (**a**–**h**) Expression patterns of selected genes from the top 50 downregulated genes. Error bars indicate standard deviation.

To further explore the core genes involved in photoperiod sensitivity, we examined the rhythmic expression profiles of selected genes from the top 50 downregulated genes (genes induced by LD condition) identified by PPI analysis (Figure 7). qRT-PCR results confirmed that these genes exhibited higher expression peaks under LD compared to SD, consistent with the RNA-seq data. This alignment between RNA-seq and qRT-PCR findings supports the reliability of the transcriptome analysis and highlights these genes as potential regulators of photoperiodic responses.

Together, these results demonstrate that photoperiod-sensitive genes, particularly those involved in circadian rhythms and photoreception, exhibit dynamic transcriptional responses to changes in day length. Phase shifts and differential expression patterns observed in LD and SD conditions underscore the role of circadian clock components and photoreceptors in regulating photoperiod adaptation. Additionally, the validation of top-ranked genes identified from PPI networks further supports their relevance in photoperiod regulation. These findings lay a foundation for future functional characterization of candidate genes involved in light signaling and flowering time regulation in maize.

## 4. Discussion

In this research, we investigated the photoperiodic response of the tropical maize inbred line Su65 under controlled LD and SD conditions. Our results demonstrated that Su65 exhibits significant photoperiod sensitivity, with SD treatment accelerating flowering time compared to LD conditions. To uncover the molecular mechanisms underlying this response, we integrated transcriptome analyses, functional enrichment assessments, and PPI network analyses. These approaches collectively highlighted the roles of key biological pathways, stress responses, and regulatory networks in photoperiod adaptation.

### 4.1. Transcriptional Adaptations to Different Between Inbreeding Lines

Transcriptome analysis has been widely used to investigate the molecular mechanisms underlying photoperiod responses in maize [26,39,40]. However, earlier studies focused on comparison between inbreeding lines with different photoperiodic sensitivity, whereas our experimental design integrated samples collected at multiple time points within 24 h under contrasting photoperiod conditions aiming to capture broader transcriptional dynamics influenced by photoperiod changes.

Previous RNA-seq studies have revealed that photoperiodic regulation influences various biological processes, including photosynthesis, phenylalanine metabolism, phosphate ion transport, and the circadian rhythm [26]. Our research used an elite tropical line Su65 as a research object compared to previous research. In RNA-seq, functional enrichment analysis revealed that many DEGs were involved in stress responses, redox regulation, and metabolic processes. GO terms such as “response to heat”, “oxidation-reduction process”, and “protein folding” underscore the adaptive strategies employed by Su65 were different from tropical lines used in previous research.

In particular, genes encoding heat shock proteins and chaperones were significantly upregulated, suggesting a protective role in maintaining protein stability under LD conditions. These findings are consistent with earlier studies demonstrating that photoperiod-induced stress responses contribute to developmental plasticity in plants.

Additionally, KEGG analysis highlighted the metabolic pathways related to lipid metabolism and phenylpropanoid biosynthesis, both of which are essential for maintaining cellular homeostasis and enabling rapid responses to environmental cues. The enrichment of pathways associated with secondary metabolite biosynthesis and membrane dynamics indicates that metabolic adjustments may be critical for photoperiod adaptation. These results provide insights into the molecular basis of photoperiod-dependent regulatory networks.

### 4.2. PPI Networks and Functional Hubs

Protein–protein interaction (PPI) analysis has proven to be a robust approach in transcriptome research, facilitating the identification of functional modules and key regulatory genes [41,42]. In this study, PPI network analysis was conducted to enhance the understanding of photoperiod-responsive genes. Upregulated DEGs, including those encoding heat shock proteins, chaperones, and stress-related factors, formed tightly connected interaction clusters. These clusters emphasize the importance of protein stability and cellular stress responses in photoperiod adaptation. Heat shock proteins and chaperones likely play critical roles in mitigating oxidative stress and maintaining cellular homeostasis under SD conditions, highlighting their functional relevance in adaptive mechanisms.

Furthermore, previous studies have highlighted the important roles of heat shock proteins in temperature-regulated flowering time in plants [43]. However, their involvement in photoperiod-regulated flowering remains inadequately explored. PPI analysis identified extensive enrichment of heat shock protein-encoding genes among the top-ranked upregulated DEGs. This observation indicates that heat shock proteins may play a crucial role in the regulation of flowering time in response to photoperiod signals.

Conversely, downregulated DEGs were enriched in pathways associated with chloroplast and mitochondrial functions, such as photosynthesis, RNA processing, and oxidative stress regulation. Hub genes, including superoxide dismutase and chloroplast RNA processing factors, were identified as the key regulators of redox balance and transcriptional activity under LD conditions. These findings suggest that photoperiod adaptation involves coordinated adjustments in metabolic processes and stress responses to optimize energy utilization and developmental timing.

PPI network analysis highlights distinct functional modules and hub genes associated with photoperiod regulation. The identification of stress-related factors, such as heat shock proteins and redox regulators, underscores their roles in enabling maize to adapt to changing day lengths. These results provide a framework for future studies aimed at validating key genes and deciphering their precise roles in photoperiod-regulated pathways.

### 4.3. Circadian Rhythm Regulation and Photoperiod Response

The circadian clock plays an important role in regulating photoperiodic responses, mediating the precise timing of gene expression in response to environmental light signals. Previous studies have identified key circadian clock components, such as *ZmFKF2/ZmFKF1b* [44], *ZmGI2* [45], and *ZmPRR37a* [24], as the central regulators of flowering time and stress adaptation in maize. These genes act within interconnected feedback loops, integrating light and photoperiod cues to synchronize physiological processes with environmental changes.

In this study, we investigated the temporal expression patterns of circadian rhythm-related genes under long-day (LD) and short-day (SD) conditions. Photoreceptor genes, including *ZmPHYB1* and *ZmPHYC1*, exhibited elevated expression levels during the 8–20 h interval under LD compared to SD, underscoring their roles in light signal perception and downstream photoperiodic regulation. In contrast, *ZmCRY4*, a cryptochrome gene, maintained consistent expression across both conditions, suggesting a photoperiod-independent function, potentially involved in basal light signal transduction. Notably, the expression patterns of *ZmPHYB1* and *ZmPHYC1* observed in this study are consistent with previous reports [20], further supporting their important role in photoperiodic regulation. Clock-associated genes, such as *ZmFKF2*, *ZmGI2*, *ZmCCT11*, and *ZmPRR37a*, displayed notable phase delay under SD conditions, indicating that SD modulates the circadian clock’s oscillatory behavior. This phase delay is in accordance with previous research indicating that *ZmPHYB1*, *ZmPHYC1*, *ZmFKF2*, *ZmGI2*, *ZmCCT11*, and *ZmPRR37a* contribute to the photoperiodic sensitivity of Su65. Furthermore, *ZmFKF2* and *COLORLESS2* showed slightly higher peak expression levels under SD, suggesting that these genes may integrate light-duration signals to optimize photoperiod adaptation.

Additionally, several key downregulated genes identified from PPI network analysis exhibited higher expression peaks under LD conditions, consistent with their roles in regulating circadian rhythms and photoperiod adaptation (as shown in Figure 7). These genes, enriched in pathways related to redox regulation and energy metabolism, likely contribute to stress responses and metabolic adjustments under varying day lengths.

Collectively, our findings reinforce the critical role of circadian rhythm regulation in photoperiod responsiveness. Genes involved in light signal perception, circadian phase modulation, and downstream metabolic processes operate in concert to coordinate developmental timing and environmental adaptation. Further functional studies targeting these genes will enhance our understanding of their roles in photoperiod regulation and may provide valuable insights for improving maize breeding strategies aimed at optimizing flowering time and stress resilience under diverse photoperiodic environments.

## 5. Conclusions

In conclusion, in this study, a comprehensive analysis is provided of the molecular mechanisms underlying photoperiod sensitivity in tropical maize. The identification of key regulatory networks, including circadian rhythm regulation, heat shock proteins, and metabolic pathways, offers valuable insights into the genetic basis of photoperiod adaptability in maize. These findings may contribute to improving maize breeding strategies, enabling the development of varieties with enhanced stress resilience and optimized flowering time under diverse environmental conditions.

## Figures and Tables

**Table 1:** Data filtering statistics under LD and SD conditions.

Sample	Raw Data	Clean Data (%)	Adapter (%)	Low Quality (%)	polyA (%)	N (%)
LD6L-A	41,666,916	41,586,194 (99.81%)	10,944 (0.03%)	65,674 (0.16%)	0 (0.00%)	4104 (0.01%)
LD6L-B	57,187,782	57,101,052 (99.85%)	12,596 (0.02%)	68,798 (0.12%)	0 (0.00%)	5336 (0.01%)
LD6L-C	58,029,652	57,941,474 (99.85%)	13,298 (0.02%)	69,426 (0.12%)	0 (0.00%)	5454 (0.01%)
SD6L-A	48,440,634	48,378,550 (99.87%)	9908 (0.02%)	47,676 (0.10%)	0 (0.00%)	4500 (0.01%)
SD6L-B	39,735,378	39,663,830 (99.82%)	10,208 (0.03%)	57,562 (0.14%)	0 (0.00%)	3778 (0.01%)
SD6L-C	57,629,566	57,530,366 (99.83%)	14,822 (0.03%)	79,038 (0.14%)	0 (0.00%)	5340 (0.01%)

Raw data represent total reads, while clean data (%) indicate high-quality reads retained post-filtering. Adapter (%), low quality (%), polyA (%), and N (%) denote reads removed due to adapter contamination, low quality, homopolymer stretches, and ambiguous bases, respectively. Each condition includes three biological replicates (e.g., LD6L-A, LD6L-B, LD6L-C).

**Table 2:** Summary of sequence data and quality statistics.

Sample	Raw Data (bp)	BF_Q20 (%)	BF_Q30 (%)	BF_N (%)	BF_GC (%)	N (%)	Clean Data (bp)	AF_Q20 (%)
LD6L-A	6,250,037,400	6,166,177,264 (98.66%)	5,972,838,712 (95.56%)	118,506 (0.00%)	3,419,480,127 (54.71%)	4104 (0.01%)	6,227,271,167	6,148,206,236 (98.73%)
LD6L-B	8,578,167,300	8,456,263,746 (98.58%)	8,175,122,750 (95.30%)	157,387 (0.00%)	4,688,750,443 (54.66%)	5336 (0.01%)	8,548,075,458	8,431,916,606 (98.64%)
LD6L-C	8,704,447,800	8,585,799,590 (98.64%)	8,308,379,400 (95.45%)	162,598 (0.00%)	4,767,978,704 (54.78%)	5454 (0.01%)	8,674,577,605	8,561,576,749 (98.70%)
SD6L-A	7,266,095,100	7,173,565,052 (98.73%)	6,949,964,263 (95.65%)	133,284 (0.00%)	4,019,863,353 (55.32%)	4500 (0.01%)	7,242,379,312	7,153,873,763 (98.78%)
SD6L-B	5,960,306,700	5,874,698,767 (98.56%)	5,679,089,362 (95.28%)	110,179 (0.00%)	3,227,758,774 (54.15%)	3778 (0.01%)	5,936,156,284	5,855,153,140 (98.64%)
SD6L-C	8,644,434,900	8,527,699,843 (98.65%)	8,257,814,731 (95.53%)	157,124 (0.00%)	4,694,489,173 (54.31%)	5340 (0.01%)	8,608,457,893	8,498,292,904 (98.72%)

Raw data represent total base pairs (bp). BF_Q20 (%) and BF_Q30 (%) denote the percentages of bases with quality scores ≥ 20 and ≥30 before filtering. BF_N (%) and BF_GC (%) represent the percentages of ambiguous bases (N) and GC content, respectively. N (%) indicates the proportion of ambiguous bases. Clean data (bp) and AF_Q20 (%) show total bases and Q20 scores after filtering.

**Table 3:** Genome mapping statistics of effective reads.

Sample	Total	Unmapped (%)	Unique_Mapped (%)	Multiple_Mapped (%)	Total_Mapped (%)
LD6L-A	40,875,198	3,438,639 (8.41%)	36,177,760 (88.51%)	1,258,799 (3.08%)	37,436,559 (91.59%)
LD6L-B	56,082,242	4,923,936 (8.78%)	49,358,514 (88.01%)	1,799,792 (3.21%)	51,158,306 (91.22%)
LD6L-C	56,555,726	4,798,584 (8.48%)	49,931,206 (88.29%)	1,825,936 (3.23%)	51,757,142 (91.52%)
SD6L-A	47,178,598	4,118,727 (8.73%)	41,628,598 (88.24%)	1,431,273 (3.03%)	43,059,871 (91.27%)
SD6L-B	38,873,086	3,730,658 (9.60%)	33,949,742 (87.33%)	1,192,686 (3.07%)	35,142,428 (90.40%)
SD6L-C	56,040,042	6,795,710 (12.13%)	47,555,238 (84.86%)	1,689,094 (3.01%)	49,244,332 (87.87%)

Total indicates the number of effective reads. Unmapped (%), Unique_Mapped (%), and Multiple_Mapped (%) denote the proportions of reads unmapped, uniquely mapped, and mapped to multiple loci, respectively. Total_Mapped (%) represents the overall mapping rate.

## Data Availability

The raw Illumina sequence reads have been deposited into the National Center for Biotechnology Information under sequence read archive (SRA) PRJNA1205870.

## Supplementary Materials

The following supporting information can be downloaded at: https://www.mdpi.com/article/10.3390/genes16020192/s1, Table S1: List of Primer sequence for qRT-PCR assay. Table S2: rRNA Read Filtering Summary. Table S3: Summary of PCA analysis. Table S4: DEGs data with classification information. Table S5: Functional Annotation and Cluster Classification of the Top 50 Upregulated Genes Based on PPI Network Analysis. Table S6: Functional Annotation and Cluster Classification of the Top 50 Downregulated Genes Based on PPI Network Analysis. Table S7: Details information of circadian rhythm-related DEGs identified from RNA-seq. Figure S1: DEG statistics under LD and SD conditions.
