# Supplementary figures and images for: Unveiling Photoperiod-Responsive Regulatory Networks in Tropical Maize Through Transcriptome Analysis
Source: Genes (Basel). 2025 Feb 4;16(2):192. doi: 10.3390/genes16020192 (PMC11855818; doi:10.3390/genes16020192)

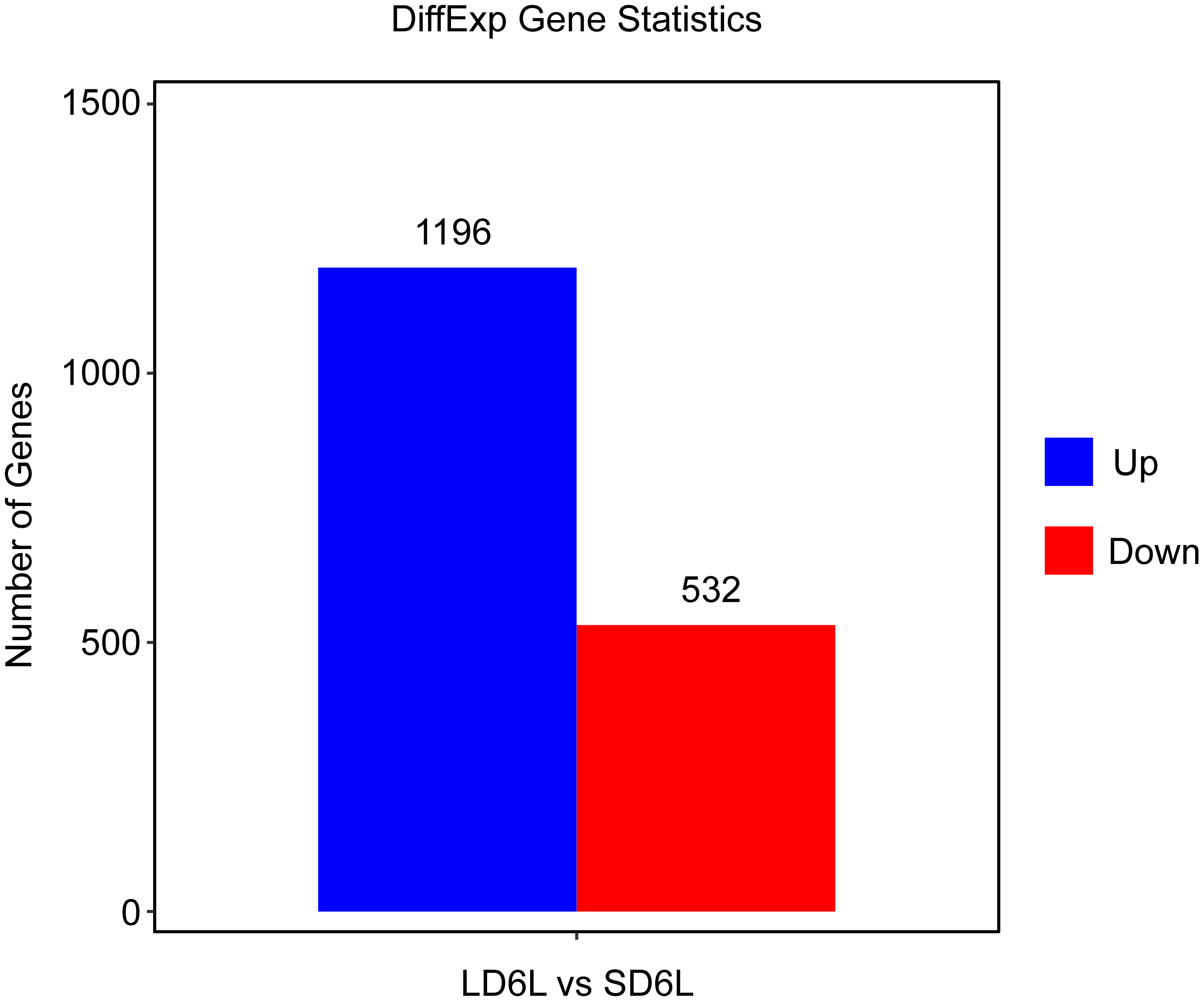

Supplement: Supplementary file 1 [file genes-16-00192-s001.zip › Figure S1.tif]
